# Case report: Incomplete bypass ileocolostomy without partial typhlectomy in five horses with acute, non-reducible cecocolic intussusceptions and review of literature

**DOI:** 10.3389/fvets.2024.1450395

**Published:** 2024-11-27

**Authors:** Antonia Troillet, Doreen Scharner

**Affiliations:** Department for Horses, Faculty of Veterinary Medicine, University of Leipzig, Leipzig, Germany

**Keywords:** horse, cecum, cecocolic, Intussusception, abdominal surgery, incomplete bypass, cecal amputation, partial typhlectomy

## Abstract

Cecocolic intussusceptions are a rare condition of acute colic in horses requiring immediate surgical intervention due to persistent uncontrollable pain and ongoing ischemic cecal necrosis. Particularly in cases where reduction of the intussusception is surgically not feasible surgical interventions such as partial typhlectomy through colotomy (partial cecal amputation) combined with or without cecal bypass techniques are described. Alternatively, surgical interventions can also be performed without partial typhlectomy via incomplete bypass ileocolostomy. Information regarding applicable techniques and outcomes base on sparse literature of single case reports or small case series. Therefore, this case series aims to add more cases treated with incomplete bypass ileocolostomy without typhlectomy to existing literature and to compare the outcome by reviewing medical records from January 2009 to March 2024 in context to literature. Five horses were surgically treated and were followed-up between 1 and 9 years. Minor short-term complications were recorded during hospitalization such as transient mild colic and febrile episodes. Long-term outcome revealed that horses received or exceed their previous level of use. By adding the hereby presented cases to published data horses treated with ileocolostomy without partial typhlectomy had a long-term survival rate of 100%. However, numbers of published cases are still low with 49 horses being included in the literature review whereof 42 recovered from surgery. The overall long-term survival rate was 53%. The added value of this study is based on the comprehensive documentation of a cohort of five horses successfully treated with an incomplete bypass procedure, demonstrating favorable long-term outcomes. Furthermore, the study advances the surgical technique by implementing the closure of mesenteric gap. The evidence for the application of the surgical technique has been strengthened.

## 1 Introduction

Cecocolic intussusception (CCI) is an uncommon cause of colic in horses with a reported prevalence of 0.01–1.9% in the majority of reports (1–6) and 14% in one report from New Zealand (7). Surgical intervention poses significant challenges in situations where the intussusception cannot be manually reduced. Several surgical options are published for non-reducible CCI in horses. Partial typhlectomy (partial cecal amputation) through right ventral colotomy is the most often reported technique aiming at surgical reduction of the intussusception and resection of the necrotic cecal intussusceptum (3–5, 7–13). This technique can be combined with either complete (9–11) or incomplete (10) bypass ileo- or jejunocolostomy. However, single reports propose an alternative option by describing complete (6, 14) or incomplete (15) cecal bypass techniques without surgical reduction of the cecal intussusception leaving the non-reducible intussusceptum *in-situ*.

Information regarding complications and outcome of the respective techniques can only be derived from low case numbers and some reports lack information regarding a detailed description of the surgical technique used, a thorough follow-up or do not mention reasons for the horses‘ early death (1, 2). Interventions involving partial typhlectomy are characterized by their invasiveness and the heightened risk of intraoperative contamination. Additionally, the combination of typhlectomy and bypass may prolong the duration of surgery and is considered one of the most technically demanding procedures in equine surgery.

To evaluate long-term outcomes in regard to the different surgical techniques objectively, we conducted a literature review and included the hereby presented cases which were treated surgically between January 2009 and March 2024 due to non-reducible CCI with incomplete bypass ileocolostomy without partial typhlectomy. This technique has been documented for only three cases so far with convincing survival rates in the long-term (15). However, critical concerns regarding the safety of the surgical technique were also mentioned (16). Therefore, the necessity to add more cases to literature seems justifiable. We included cases where the diagnosis of a non-reducible CCI was confirmed by exploratory laparotomy and when interventional surgery was desired by the owner. Signalment, pre-surgical findings, surgical technique as well as post-operative management and outcome were documented. Follow-up was assessed by telephone or e-mail consultation of owners or referring veterinarians with a minimum of 6 months after discharge and continued on a regularly base until horses were lost for further follow-up. Survival analysis was performed using Kaplan–Meier method. 95% confidence intervals were calculated for short- and long-term survival. Log rank test was used to statistically compare the survival curves and to compute *p*-values. Level of significance was set at *p* < 0.05.

## 2 Case description

### 2.1 Surgical technique

**Figure 1:**
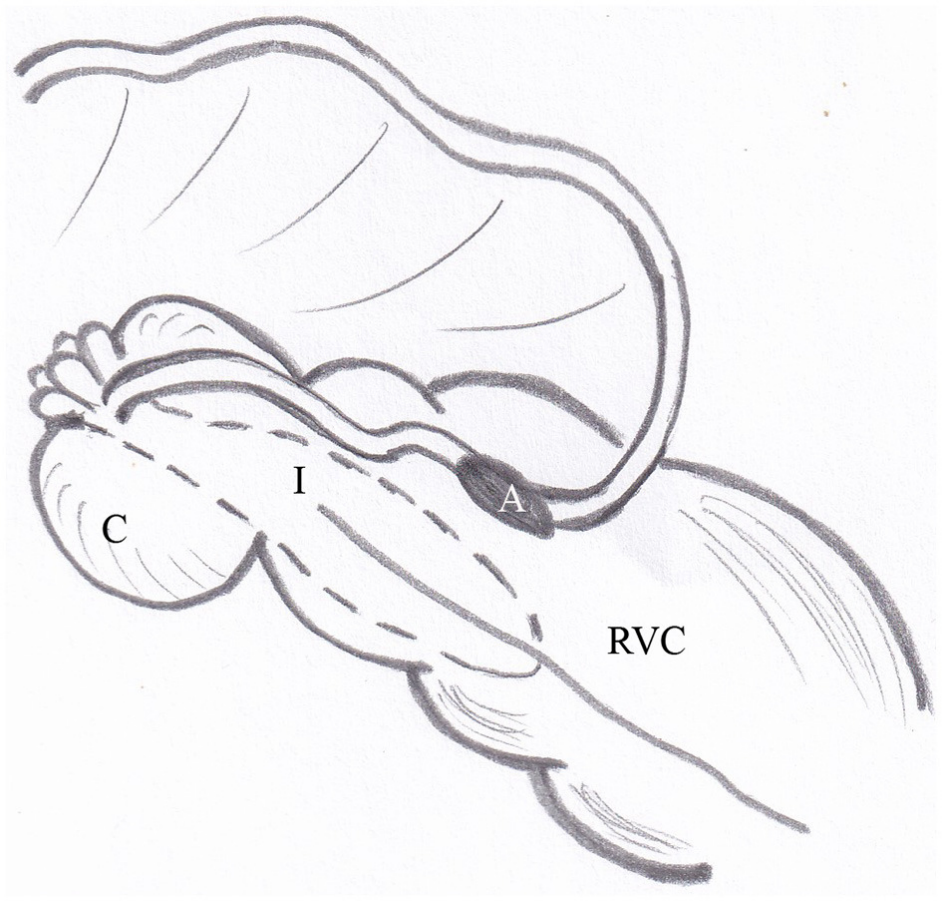
Illustration of the surgical technique of incomplete bypass ileocolostomy. The side-to-side anastomosis of ileum und colon is positioned with the ileum in anti-peristaltic orientation between the ventrolateral and ventromedial taenia of the ventral colon at its most orally exteriorized portion. A, anastomosis; C, cecal stump; I, intussusceptum; RVC, right ventral colon.

The surgical technique complies with the previously published technique (15). Incomplete bypass ileocolostomy was performed as an approximate 10-cm side-to-side hand-sewed anastomosis of two layers. The first layer performed as a full thickness suture in single continuous pattern followed by a second seromuscular Cushing-pattern using 2-0 polyglactin 910. The ileal-colonic orientation was set anti-peristaltic with the ileum positioned between the two free bands (ventrolateral and ventromedial) at the most orally exteriorized portion of the ventral colon (Figure 1). In addition to the published technique, closure of the gap between the ileocecal fold, cecocolic fold and colon serosa was performed with simple continuous patterns using 2-0 polyglactin 910 in order to reduce the risk of intestinal entrapment. The intussusception was not oversewed nor manipulated further.

### 2.2 Signalment and pre-surgical findings

Within the defined period five horses met the inclusion criteria (Table 1). Age ranged from 2 to 11 years (median 7). Four mares and one gelding represented the following breeds: 3 Ponies, 1 Quarter Horse, 1 Warmblood. Duration of clinical signs of colic prior to admission to the hospital ranged from 6 to 24 h (median 12). None of the horses had a history of previous colic episodes. All horses had been treated medicinally without appropriate response by the referring veterinarian. Information about anthelmintic medication regimes was available for four cases (3 regularly, 1 none). Horses were used for breeding (1), pleasure riding (2) and two horses were unbroken. All horses had an overall breed and age characteristic body condition score.

**Table 1:** Overview of signalment, clinical signs and outcome five horses treated with ileocolcostomy without cecal amputation due to a non-reducible cecocolic intussusception.

	**Signalment**				**Clinical findings at admission**						**Selected blood parameter at admission**				**Follow-up**		
**Case**	**Breed**	**Age (years)**	**Sex**	**Intended use**	**Heart rate (beats/ min)**	**Respiration rate (breaths/ min)**	**Rectal temperature (**°**C)**	**Intestinal sounds**	**Mucous membrane color**	**Capillary refill time (secs)**	**PCV (L/L)**	**TP (g/L)**	**WBC count (G/l)**	**Lactate (mmol/L)**	**Duration**	**Clinical signs**	**Level of use or exercise**
1	Shetland pony	11	Mare	Breeding	88	30	36.5	Absent	Reddened	>3	0.61	56	3.0	12.6	5 years (euthanized unrelated to colic)	Mild colic, treated medically once	Breeding
2	Sport pony	10	Mare	Pleasure	48	20	37.5	Absent	Pale pink	2	0.28	60	5.5	1.4	9 years (sold)	None	Pleasure riding
3	Pony	7	Gelding	Pleasure	52	24	38.2	Reduced	Reddened	>3	0.45	65	5.6	1.3	1 year (sold)	None	Pleasure riding
4	Quarter horse	2	Mare	Unbroken	60	24	38.0	Reduced			0.44	62	17	1.5	3 years (still under follow-up)	None	International level, western
5	Dutch Warmblood	4	Mare	Broken	90	34	n.r..	n.r.			0.30	46	n.r.	2.1	1 year (still under follow-up)	None	First ridden training level

n.r., not recorded; PVC, packed cell volume; TP, total protein; WBC, white blood cells.

At admission three horses demonstrated signs of severe abdominal pain (case 1, 2, 5), one horse showed mild abdominal pain (case 3), and one horse was depressed (case 4). Clinical findings were tachycardia (48–90/min, median 60), tachypnoea (24–34/min, median 24) and a reduced or absent intestinal motility on auscultation. Transrectal temperature was within the physiological range. All horses demonstrated reddened mucous membranes and a prolonged capillary refill time. Gastric reflux could be obtained in one horse (case 1). Transrectal palpation revealed no abnormal findings in two horses (case 2, 4). In three horses, rectal palpation was not performed due to the small size of the horse (case 1), a superficial rectal mucosa laceration (case 3) and severe uncontrollable colic signs (case 5). Transabdominal ultrasonography demonstrated the characteristic target-like large intestinal pattern on the right side of the caudoventral abdomen in two horses (case 3, 4) where a presumptive diagnosis of intussusception was made. One horse (case 1) demonstrated distended and amotile small intestinal loops along the ventral abdomen. One horse was ultrasonographical unremarkable (case 2) and one could not be examined due to the horse‘s agitation (case 5). Blood analysis at admission revealed a wide range of packet cell volume (0.28–0.61 L/L, median 0.44), total protein (46–65 g/L, median 60) and lactate 1.3–12.6 mmol/L (median 1.5). The total white blood cell count ranged from 3.0 to 17 G/l (median 5.5). Abdominocentesis was not performed. Indication for surgery based on the severe grade of pain (case 1, 2, 5), the duration of colic (case 1, 4) and the non-responsiveness to medical treatment (all). Furthermore, abnormal sonographic findings were considered indicative for surgery in three horses (case 1, 3, 4), (Table 1).

### 2.3 Specific surgical intervention

Surgery was performed as previously described in all horses. In one horse (case 2), evacuation of the small intestine was required due to severe intraluminal fluid accumulation, which was addressed through jejunal enterotomy. In another horse (case 4), the colon was emptied via pelvic flexure enterotomy before suturing the anastomosis. Horses recovered without assistance as is customary in our clinic.

### 2.4 Post-operative management

All horses recovered uneventful from anesthesia. Post-operatively horses received intravenous lactated Ringer solution and electrolyte supplementation according to hydration status under acid-base and electrolyte control. Antibiotic therapy of sodium penicillin (20,000 UI/kg IV every 8 h) and gentamicin sulfate (6.6 mg/kg IV every 24 h) was maintained for 6 to 14 days (median 9) in all horses. Additionally, one horse received metronidazole (20 mg/kg orally every 12 h) for 7 days and a plasma transfusion (case 5). Further medication included flunixin meglumine in a dose of 0.6 mg/kg IV twice daily for 6 to 14 days (median 8) and 2 % lidocaine hydrochloride in a continuous rate of 0.05 mg/kg/min administered within the first 48 h after surgery. Twenty-four hours after surgery horses were introduced to water. Hay was given in small portions upon the third post-operative day increasing in portion size. All horses received anthelmintic oral medication (praziquantel 1.5 mg/kg and 200 μg/kg ivermectin).

In addition to the standard follow-up examinations of horses that underwent colic surgery, the focus was placed on transcutaneous abdominal ultrasound assessments to evaluate small intestine motility, to document the resolution of the invagination, and to monitor signs of peritonitis, such as increased abdominal fluid. Data of repeated post-operative ultrasonographic examinations was available from two horses (case 1, 5). The first reported case was hospitalized for scientific interest to follow-up the characteristic target-like large intestinal pattern at the right caudoventral abdomen signs to be no longer reproducible up for 34 days whereas in the other case the typical ultrasonographic findings were still present until discharge at day 15.

Initially two of the five horses showed a depressed general behavior for up to 3 days after surgery (case 1, 5). During hospitalization intermittent mild to moderate colic episodes were noticed in three horses at the first post-operative day (case 2, 3) and until day 14 (case 4), These symptoms resolved after hand walking and a single dose of 2.5 mg/kg metamizole IV. A temporarily febrile rectal temperature was noted in one case (case 4) for three post-operative days. Time of hospitalization ranged from 12 to 34 days (median 15). All horses survived to hospital discharge. The abdominal wounds were unremarkable without signs of a surgical site infection at that date.

### 2.5 Outcome and literature review

Long-term follow-up from 1 to 9 years (median 3) was available for all horses. Owner reported that all horses regained their pre-surgical level of use or were broken and ridden uneventfully (Table 1). For one horse a single episode of mild and unspecific colic was reported 1 year after surgery which was treated with a single analgesic medication by the home veterinarian (case 1).

Derived from literature detailed information on applied surgical procedures for non-reducible CCI was available for 49 horses of which 42 (86%) recovered from surgery. Thirty-five horses were discharged from hospital (71%). A long-term follow-up with a minimum of 6 months was available for 26 horses, calculating an overall long-term survival rate of 53% of all surgically treated horses (Table 2). By adding the present case series to published data, incomplete bypass ileocolostomy without partial typhlectomy demonstrated a long-term 100% survival rate in 8 horses over a follow-up period of 1–9 years whereas complete bypass techniques without partial typhlectomy refer to 7 horses with a 100% discharge rate and a long-term survival rate of 57% (6, 14). Data derived from reports describing partial typhlectomy through colotomy revealed 39 horses of which 25 horses (64%) were discharged and 19 (49%) were reported to be alive for at least 6 months. No information about outcome after discharge was provided for eight horses and two horses died within the follow-up period (3–5, 7–13). Based on this data an incomplete bypass ileocolostomy without partial typhlectomy demonstrates superior survival rates than techniques including a complete bypass or partial typhlectomy. The comparison of outcomes between the surgical technique of partial typhlectomy via colotomy (with or without bypass) and the presented approach of incomplete bypass ileocolostomy without partial typhlectomy yielded a significant result (p=0.045). No significant differences in outcomes were observed between complete bypass ileo/jejunocolostomy without partial typhlectomy and the published cases of incomplete bypass ileocolostomy without partial typhlectomy in comparison to the present case series (Table 3).

**Table 2:** Literature review of horses surgically treated due to non-reducible cecocolic intussusceptions focusing on surgical technique and outcome.

**CCI to number celiotomies**	**Age and breed**	**Details and number of surgically treated non-reducible CCI**	**Outcome: number horses and duration**	**Tapeworm evidence in CCI**	**Reference**
**Partial typhlectomy through colotomy (with and w/o bypass)**					
2/216 (1.9%)	16 y Hunter	Total sx 1 Partial typhlectomy through colotomy (1/1)	0/1 finished sx	2/2	(3)
n.r.	1 y TB	Total sx 1 Partial typhlectomy through colotomy and ileocolostomy (1/1)	1/1 discharged long-term n.r.	n.r.	(8)
n.r.	1 y FR	Total sx 1 Partial typhlectomy through colotomy and oversew point of invagination (1/1)	1/1 discharged 1/1 follow-up 9 mo	n.r.	(12)
n.r.	2 y TB	Total sx 1 Partial typhlectomy through colotomy and oversew point of invagination (1/1)	1/1 discharged 1/1 follow-up 6 mo	n.r.	(13)
n.r.	2 y SB	Total 1 sx Partial typhlectomy through colotomy and oversew point of invagination and complete bypass ileocolostomy (1/1)	1/1 discharged 1/1 follow-up 1 y	1/1	(11)
4/310 (1.3%)	2–10 y Pony (3), TB-Mix (1)	Total 4 sx Dead during induction (1/4) Partial typhlectomy through colotomy (3/3) and Complete bypass jejunocolostomy (1/3)	1/3 finished sx 0/3 to discharge	1/4	(4)
11/842 (1.3%)	7 mo−8 y 7 SB, 3 TB	Total 4 sx Partial typhlectomy through colotomy (3/4) Ileocolostomy and oversewing cecal base in 2^nd^ sx (1/4)	0/3 discharged 1/1 discharged 1/1 died 3 wks post sx due to colic	8/10	(5)
19/135 (14%)	8 mo−12 y Breed n.r.	Total 7 sx Partial typhlectomy through colotomy (7/7)	4/7 finished sx 3/7 discharge long-term n.r.	n.r.	(7)
n.r.	1–8 y Breed n.r.	Total 8 sx Partial typhlectomy through colotomy (8/8)	8/8 discharged 1/8 died 3 mo post sx colic 7/8 follow-up 6-96 mo	6/8	(9)
n.r.	7 mo−30 y Mainly SB	Total 11 sx Partial typhlectomy through colotomy (9/11) Complete bypass ileocolostomy (2/11)	9/9 discharged 0/2 discharged 9/11 follow-up 12 mo	15/30	(10)
**Complete bypass w/o partial typhlectomy**					
n.r.	1 y TB	Total 1 sx Complete bypass ileocolostomy w/o partial typhlectomy (1)	1/1 discharged 1/1 follow-up 4 mo	n.r.	(14)
8/541 (1.48%)	6 mo−6 y WB (2), Pony (2), SB (1), L (1)	Total 6 sx Complete bypass ileocolostomy w/o partial typhlectomy (1/6) Complete bypass jejunocolostomy w/o partial typhlectomy (5/6)	6/6 discharged 2/6 survived 2.5 mo 4/6 follow-up 1–7.5 y	n.r.	(6)
**Incomplete bypass w/o partial typhlectomy**					
n.r.	2 y, 1 y SB (3)	Total 3 sx Incomplete bypass ileocolostomy w/o typhlectomy (3/3)	3/3 discharged 3/3 follow-up 12 mo	n.r.	(15)

mo, months; n.r., not recorded; sx, surgery; w/o, without; wks, weeks; y, years; CCI, cecocolic intussusception; FT, Foxtrotter; FR, Frisian; L, Lipizzaner; SaB, Saddlebred; SB, Standardbred; TB, Thoroughbred.

**Table 3:** Summary of literature review and presented cases regarding short- and long-term outcome.

	**Surgical techniques and number of horses**	**Short-term outcome (discharged horses)**	**Long-term outcome (>6 mo follow-up)**

**Literature review**	Partial typhlectomy through colotomy with and w/o bypass: 39	25 (64%) (95% CI [47,2-78,3%])	19 (49%) (95% CI [32,7-65%])
	Complete bypass ileo/jejunocolocstomy w/o partial typhlectomy: 7	7 (100%) (95% CI [56,1-100%])	4 (57%) (95% CI [11,8-79,8%])
	Incomplete bypass ileocolostomy w/o partial typhlectomy: 3	3 (100%) (95% CI [31,0-100%])	3 (100%) (95% CI [31,0-100%])
	**Total number sx: 49 Recovered horses: 42**		
**Presented case series**	Incomplete bypass ileocolostomy w/o partial typhlectomy: 5	5 (100%) (95% CI [46,3-100%])	5 (100%) (95% CI [46,3-100%])
	**Total number sx: 5 Recovered horses: 5**		

CI, confidence interval; mo, months; sx, surgery; w/o, without.

## 3 Discussion

The presented case series adds five cases to the veterinary knowledge of a rare colic-related condition in horses. The authors consider publication of this case series as important for equine surgeons because descriptions of surgical interventions vary and in the majority of reports partial typhlectomy through colotomy is included in the procedure. When summarizing all published data, the incomplete bypass technique without partial typhlectomy should be seriously considered as a feasible and straight-forward alternative technique with an excellent outcome though the number of cases is still comparatively low.

In all presented horses, surgery was performed within a short duration after the onset of colic signs. Even then, attempts to reduce the intussusception manually for ~10 min were unsuccessful. In consequence, the described surgical intervention was applied. It is the authors opinion that leaving the intussusception *in-situ* allows a less invasive surgical procedure, reduces the risk of intra-operative contamination and seems time-saving. However, definite comparison of surgery times would require such data from other techniques and therefore this remains an author‘s statement. A very early report already mentioned the surgical technique that aims on an intraluminal necrotizing procedure by oversewing the cecal intussusception (17). Some authors performed oversewing of the cecal stump after partial thyphlectomy but an improvement of safety seems questionable as the value of the procedure cannot be derived from published data (5, 11–13). None of the presented and reported horses treated without partial typhlectomy developed clinical and ultrasonographic signs of septic peritonitis nor signs of adhesion formation post-operatively (6, 14, 15). It can be assumed that non-manipulation the CCI gradually leads to intraluminal adhesion formation and retraction of the remaining cecal stump. The mentioned intraluminal necrotizing process can be followed by the typical ultrasonographic appearance of the intussusception which was still visible at day 15 in one horse but not at day 34 in another. Also, transient mild colic symptoms and febrile episodes, which were noticed in three cases during hospitalization, might be related to the involution procedure of the intussusception. Systemic antimicrobial coverage for a duration of 6 days up to 2 weeks and anti-inflammatory medication based on clinical signs, laboratory and abdominal ultrasonographic findings and are comparable to other reports not including partial typhlectomy (6, 14, 15).

CCI affected horses demonstrate a rather young horse population in some reports which is in contrast to the presented cases where only one horse had an age of < 3 years (5, 8–13). All five horses in this study were admitted in an acute stage of colic with a duration of ≤ 24 h. Subclinical and chronic courses of the disease are also reported (6, 7, 9, 10). However, although in the disease‘s etiopathology tapeworm (*Anoplocephala perfoliata*) infection demonstrates strong evidence, no clear statement can be drawn whether tapeworm infections correlate to the stage of clinical signs. Summarizing data provided by literature, tapeworm infections were detected in 60% of CCI affected horses (3–5, 9–11). As the cecal intussusception was not manipulated in the presented study no information regarding intraluminal tapeworm occurrence can be provided. A final statement on tapeworm prevalence is therefore not possible since a negative fecal analysis is regarded unreliable (18).

The grade of pain, the non-responsiveness to medication and abnormal sonographic findings were indications for performing surgery. However, setting a presumptive diagnosis of a CCI was possible in two horses in the present study considering that one horse demonstrated amotile intestinal loops and one case was excluded due to severe colic signs, thus concluding one unremarkable or misdiagnosed case. The value of transabdominal ultrasonographic examination for pre-operative diagnosis has been already highlighted by other authors (6, 7, 10, 15). Another indicative finding is a transrectal palpable firm mass at the right dorsal/caudal abdomen (2, 5–7, 9, 12–14). Unremarkable or unspecific rectal findings like documented for the two assessable horses in the present study are also described (1, 2, 5, 6, 9, 11, 15). It is assumed that in subacute and chronic cases the typical rectal palpable mass can be found more frequently (10).

Literature research revealed the difficulty to assess case series with precise descriptions of the surgical technique used as well as the clear definition of CCI being either reducible or not. Therefore, two reports describing 1 and 9 horses respectively were not included in the review although information regarding the incidence and clinical signs of CCI in horses are provided (1, 2). The literature review confirms that CCI has to be considered as a rare condition in horses and illustrates that in non-reducible cases surgical intervention carries an overall guarded prognosis. The comparable low number of horses treated with incomplete bypass ileocolostomy without partial tyhphlectomy is a clear limitation of the study, however a 100% long-term survival rate stated by two independent reports is considered promising to increase application in equine surgery. The publication of even small case numbers appears urgently necessary for such a rare disease in order to further investigate the evidence of the surgical techniques.

## Data Availability

The original contributions presented in the study are included in the article/supplementary material, further inquiries can be directed to the corresponding author.
